# Comprehensive analysis of cuproptosis-related genes in immune infiltration in ischemic stroke

**DOI:** 10.3389/fneur.2022.1077178

**Published:** 2023-02-02

**Authors:** Xuehui Fan, Hongping Chen, Fangchao Jiang, Chen Xu, Yingju Wang, Haining Wang, Meng Li, Wan Wei, Jihe Song, Di Zhong, Guozhong Li

**Affiliations:** Department of Neurology, The First Affiliated Hospital of Harbin Medical University, Harbin, Heilongjiang, China

**Keywords:** cuproptosis, ischemic stroke, immune infiltration, risk model construction, predictors

## Abstract

**Background:**

Immune infiltration plays an important role in the course of ischemic stroke (IS) progression. Cuproptosis is a newly discovered form of programmed cell death. To date, no studies on the mechanisms by which cuproptosis-related genes regulate immune infiltration in IS have been reported.

**Methods:**

IS-related microarray datasets were retrieved from the Gene Expression Omnibus (GEO) database and standardized. Immune infiltration was extracted and quantified based on the processed gene expression matrix. The differences between the IS group and the normal group as well as the correlation between the infiltrating immune cells and their functions were analyzed. The cuproptosis-related DEGs most related to immunity were screened out, and the risk model was constructed. Finally, Gene Ontology (GO) function, Kyoto Encyclopedia of Genes and Genomes (KEGG) pathway enrichment analyses and drug target were performed using the Enrichr website database. miRNAs were predicted using FunRich software. Finally, cuproptosis-related differentially expressed genes (DEGs) in IS samples were typed, and Gene Set Variation Analysis (GSVA) was used to analyze the differences in biological functions among the different types.

**Results:**

Seven Cuproptosis-related DEGs were obtained by merging the GSE16561 and GSE37587 datasets. Correlation analysis of the immune cells showed that NLRP3, NFE2L2, ATP7A, LIPT1, GLS, and MTF1 were significantly correlated with immune cells. Subsequently, these six genes were included in the risk study, and the risk prediction model was constructed to calculate the total score to analyze the risk probability of the IS group. KEGG analysis showed that the genes were mainly enriched in the following two pathways: D-glutamine and D-glutamate metabolism; and lipids and atherosclerosis. Drug target prediction found that DMBA CTD 00007046 and Lithocholate TTD 00009000 were predicted to have potential therapeutic effects of candidate molecules. GSVA showed that the TGF-β signaling pathway and autophagy regulation pathways were upregulated in the subgroup with high expression of cuproptosis-related DEGs.

**Conclusions:**

NLRP3, NFE2L2, ATP7A, LIPT1, GLS and MTF1 may serve as predictors of cuproptosis and play an important role in the pathogenesis of immune infiltration in IS.

## Introduction

Stroke is the second leading cause of death in the world with 15 million new ischemic stroke (IS) cases and 6.5 million IS-related deaths every year (1). IS is a complex pathological process with a variety of mechanisms involving excitotoxicity, oxidative damage, inflammation and Ca^2+^ overload (2). Many studies have confirmed that GFAP, MBP and NSE can be used as biomarkers of IS (3). Accumulating evidence suggests that massive recruitment of immune cells plays a central role in the acute phase of stroke. Infiltrating peripheral immune cells and resident microglia mediate neuronal cell death and blood–brain barrier (BBB) disruption by releasing inflammation-related molecules (4). Therefore, exploring the pathogenesis of immune infiltration in IS may provide new ideas for reducing pathological damage after IS.

As a cofactor of essential enzymes, copper plays an important role in human life activities (5). The concentration of copper in normal cells is low, and it mainly maintains cellular copper homeostasis by preventing harmful intracellular free copper accumulation through the dynamic equilibrium mechanism across the concentration gradient (6). However, a recent study has shown that cuproptosis is different from the previously characterized forms programmed cell death (such as ferroptosis and apoptosis) as cuproptosis depends on mitochondrial respiration. In this process, copper directly binds to the lipidation components of the tricarboxylic acid cycle, resulting in the aggregation of lipoacylated proteins and the loss of iron-sulfur cluster proteins, leading to proteotoxic stress and cell death (7). The importance of copper homeostasis in immune infiltration has also been suggested in recent correlation studies. Tan et al. (8) found that copper chelation on macrophages eliminates lysyl oxidase-like 4-mediated programmed death ligand 1 presentation, thereby inhibiting the immune escape of cells. Choi et al. (9) showed that clodoxoline (a common copper chelator) effectively reduces the infiltration of immune cells (CD4^+^ and CD8^+^ T cells) that cause encephalitis. Therefore, it is of great importance to elucidate the mechanism of cuproptosis in immune infiltration. However, no study on the correlation of cuproptosis with immune infiltration and the progression of IS has been reported. The present study is the first to elucidate the specific mechanism of immune infiltration mediated by IS cuproptosis by combining IS microarray data and cuproptosis-related genes.

We used the GSE16561 and GSE37587 datasets of the GEO database to analyze the correlation and difference between immune cells and functions, and we combined them with the cuproptosis-related genes reported by Tsvetkov et al. (7) to analyze their correlation with immune cells and functions. A risk prediction model was then built. Based on the IS-related cuproptosis-related genes obtained above, Gene Ontology (GO) function analysis, Kyoto Encyclopedia of Genes and Genomes (KEGG) pathway analysis, drug target prediction and miRNA prediction were performed. Finally, the IS group was classified, and Gene Set Variation Analysis (GSVA) was performed to provide theoretical support for the study of the regulatory mechanism of IS cuproptosis.

## Methods

### Microarray data

**Figure 1:**
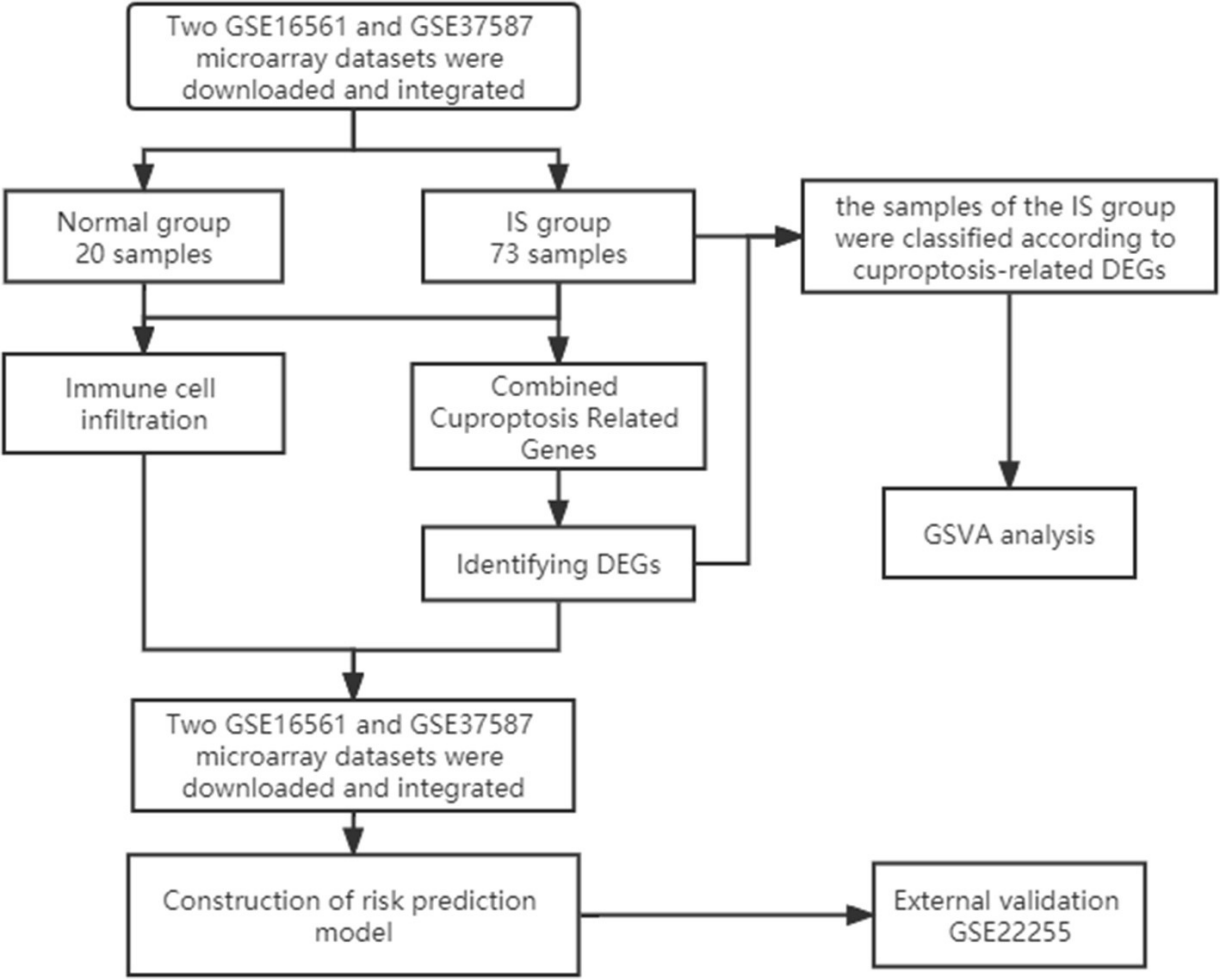
Study flow chart.

First, we downloaded two IS microarray datasets (GSE16561 and GSE37587) from the NCBI Gene Expression Omnibus (GEO) (https://www.ncbi.nlm.nih.gov/geo/). The two sample datasets were whole blood samples of human peripheral blood. Sample collection time point was within 24 h after ischemic stroke. Both datasets are from the same platform, and we identified cuproptosis-related genes from previous literature (7). The list of cuproptosis-related genes is provided in the Supplementary Data and Supplementary Figure S1A. This study requires no experiments to be conducted by any author on humans or animals. The flowchart of it is shown in Figure 1.

### Data processing and identification of cuproptosis-related DEGs

We preprocessed the two original datasets in R, including background calibration, normalization and log2 transformation (10). When multiple probes corresponded to a common gene, the average value was considered as its expression value. In addition, Bioconductor's surrogate variable analysis, “Sva” package, was used to eliminate batch effects between the two datasets (11). In the final combined dataset, there were 24 samples in the normal group and 73 samples in the IS group. Subsequently, the expression matrix of CC-related genes was obtained by combining the expression data. Finally, the “Limma” package was applied to screen DEGs.

### Immune cell infiltration analysis of cuproptosis-related DEGs

To quantify the relative proportion of infiltrating immune cells in the IS group, CIBERSORT, a method for analyzing different immune cell types in tissues, was used to analyze the pooled expression data and calculate immune cell infiltration (12). The percentage of each immune cell type in the sample was calculated and displayed in the bar graph. The levels of 22 immune cells were compared between the IS group and the normal group (13). Finally, the correlation analysis of 22 infiltrating immune cells of cuproptosis-related DEGs was displayed by a heatmap.

### Risk prediction model construction and enrichment analysis

According to the immune cell infiltration analysis of cuproptosis-related genes, the six most immune-related genes were screened out, and a risk prediction model was constructed. A nomogram was then used to predict the incidence of the disease. ROC curves were used to observe the accuracy of the models. GO analysis, KEGG pathway enrichment analysis and drug target prediction of key genes were performed through the Enrichr website (https://maayanlab.cloud/Enrichr/). Finally, gene function enrichment and interaction network analysis were performed by FunRich software.

### Sample typing and GSVA of the IS group

The “ConsensusClusterPlus” package was used for consensus analysis (14). The IS group was classified into two types according to the expression of cuproptosis-related DEGs. The heatmap shows the difference in cuproptosis-related DEGs between the different types. PCA showed that the type 1 and type 2 samples were better differentiated. Subsequently, we compared the levels of 22 immune cells between the two subtypes and used the “GSVA” package to perform GSVA to evaluate the differences in biological functions between different subtypes (15).

## Results

### Identification of cuproptosis-related DEGs

**Figure 2:**
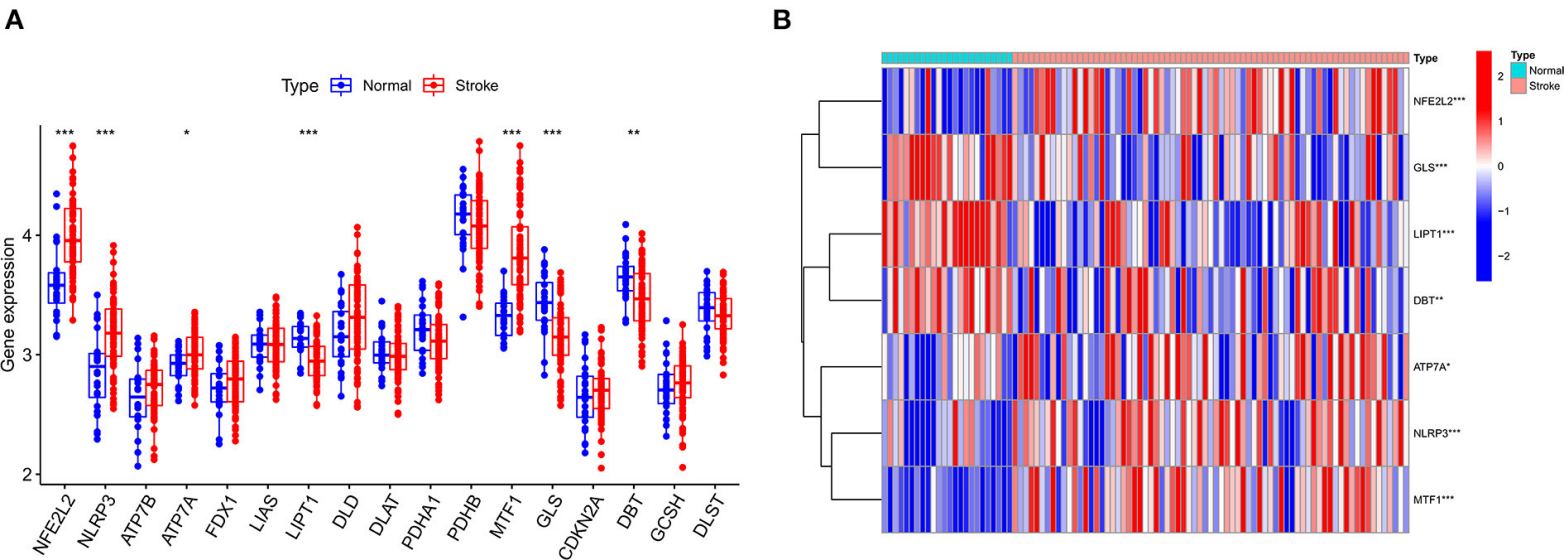
Boxplot and heatmap for differential analysis of cuproptosis-related genes after combined datasets. **(A)** For the boxplot, the abscissa is the gene associated with cuproptosis, and the ordinate is the expression of the gene. **(B)** For the heatmap, the abscissa is the sample, and the ordinate is the gene. Red represents genes with high expression, and blue represents genes with low expression. *P < 0.05, **P < 0.01 and ***P < 0.001.

**Figure 3:**
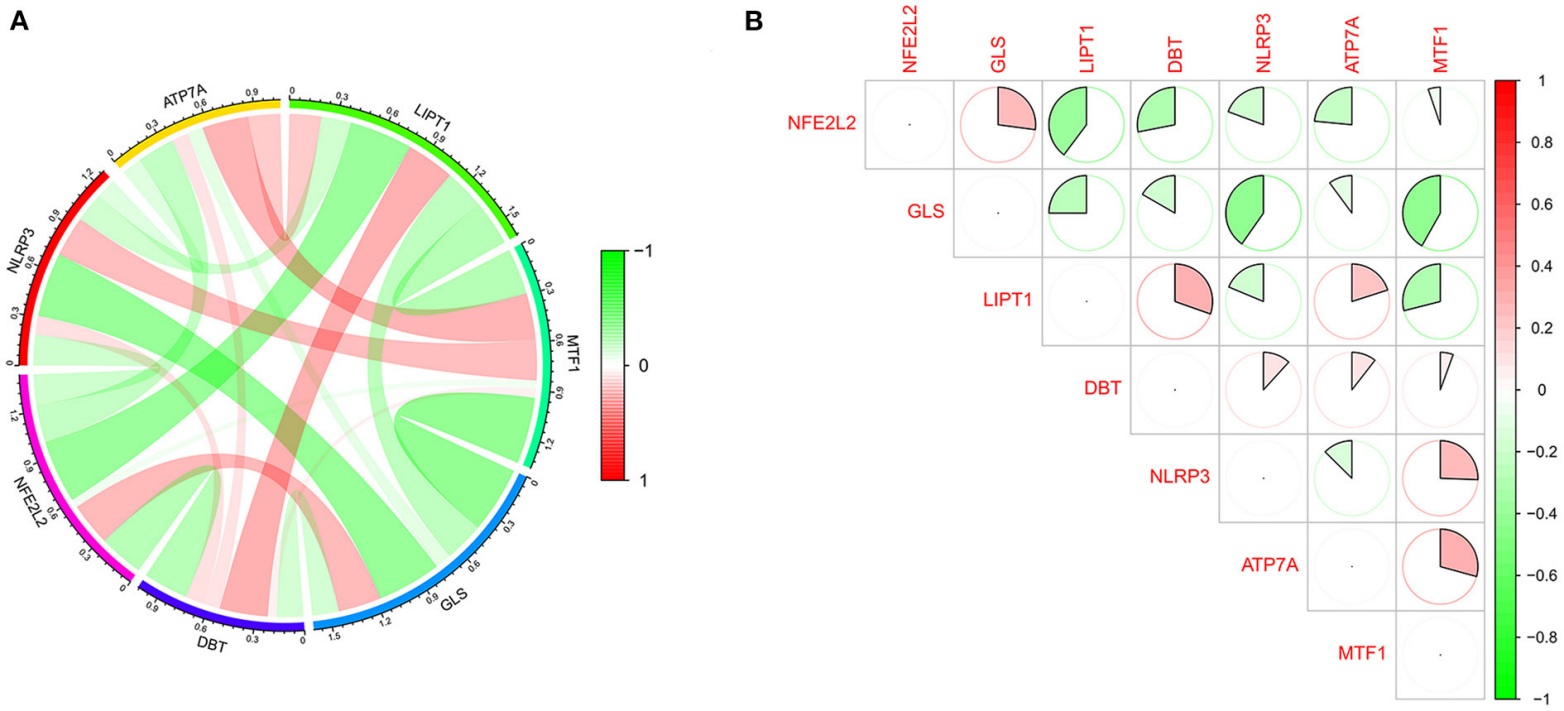
Correlation analysis of cuproptosis-related DEGs. **(A)** For the circle plot, the outermost layer represents the cuproptosis-related DEGs, and the line indicates the correlation between genes. A positive correlation is shown in red, and a negative correlation is shown in green. Darker colors represent more significant correlations. **(B)** For the matrix plot, the abscissions and diagonals are cuproptosis-related DEGs. A positive correlation is shown in red, and a negative correlation is shown in green. Larger sector areas indicate more significant correlations.

The GSE16561 and GSE37587 datasets were merged to obtain the batch corrected data, and the data expression matrix was merged with cuproptosis-related Genes to obtain the expression matrix of cuproptosis-related Genes. Seven differentially expressed genes were obtained, and the boxplots (Figure 2A) and heatmaps (Figure 2B) of differentially expressed genes were generated. In the IS group, the upregulated genes were NFE2L2, NLRP3, ATP7A and MTF1, and the downregulated genes were LIPT1, GLS and DBT. Subsequently, we conducted correlation analysis of these genes. Strong positive correlations were observed between the following pairs: NFE2L2 and GLS; LIPT1 and DBT; MTF1 and NLRP3; and MTF1 and ATP7A. Negative correlations were observed between the following pairs: NFE2L2 and LIPT1; GLS and NLRP3; and GLS and MTF1 (Figure 3).

### Analysis of immune cell infiltration

**Figure 4:**
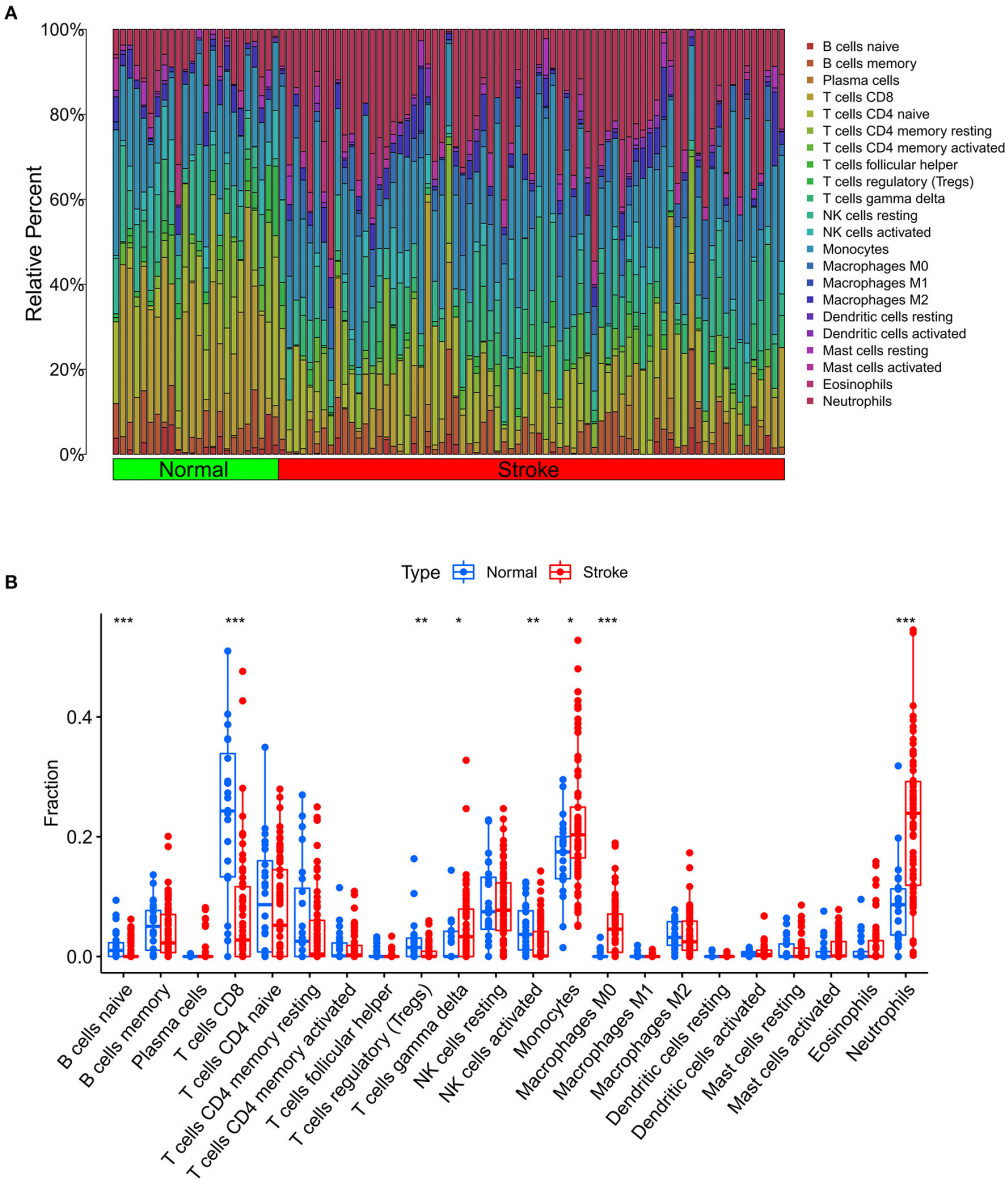
Analysis of immune infiltration in the IS group and the normal group. **(A)** Relative percentages of 22 immune cells in the IS and normal groups. **(B)** Comparison of 22 immune cell subtypes between IS and normal subjects. *P < 0.05, **P < 0.01 and ***P < 0.001.

**Figure 5:**
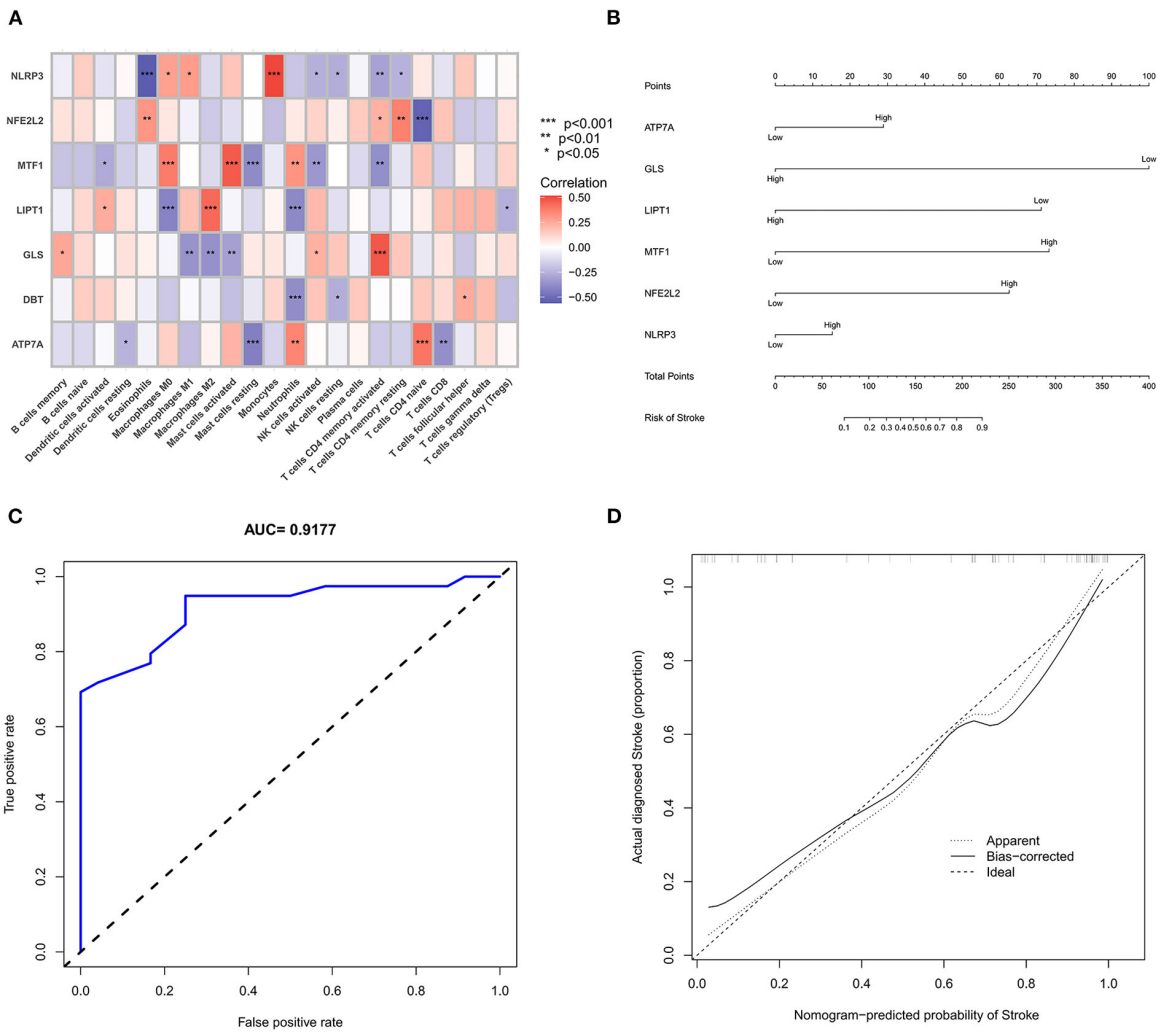
Correlation analysis and gene prediction model of cuproptosis-related DEGs and immune cells. **(A)** Correlation analysis of cuproptosis-related DEGs and immune cells. The abscissa represents the name of immune cells, and the ordinate represents the genes related to death. Red represents positive correlation, and blue represents negative correlation. Darker colors indicate stronger correlations (*P < 0.05, **P < 0.01 and ***P < 0.001). **(B)** Nomograms, which were based on the correlation analysis of immune cells, screened out the most immune-related genes to construct a risk prediction model. The IS risk index of patients was predicted through nomograms. **(C)** ROC curve. **(D)** Calibration curve.

We further used the CIBERSORT algorithm to predict immune cell infiltration between the IS and normal groups. The percentages of the 22 immune cells in each sample are shown in Figure 4A. Compared to the normal group, gamma delta T cells, monocytes, M0 macrophages and neutrophils were upregulated in the IS group, and naive B cells, CD8^+^ T cells, regulatory T cells (Tregs) and activated NK cells were downregulated in the IS group (Figure 4B). Subsequently, we conducted correlation analysis of immune cells and the DEGs, namely, NLRP3, NFE2L2, ATP7A, LIPT1, GLS and MTF1. The following pairs showed a positive correlation: NLRP3 and monocytes; MTF1 and activated mast cells; MTF1 and M2 macrophages; GLS and activated CD4^+^ memory T cells; and LIPT1 and M2 macrophages. The following pairs showed a negative correlation: NLRP3 and eosinophils; and NFE2L2 and CD4^+^ naïve T cells (Figure 5A). Therefore, NLRP3, NFE2L2, ATP7A, LIPT1, GLS and MTF1 were considered the most immune-related cuproptosis-related DEGs.

### Construction and analysis of the prediction model

**Figure 6:**
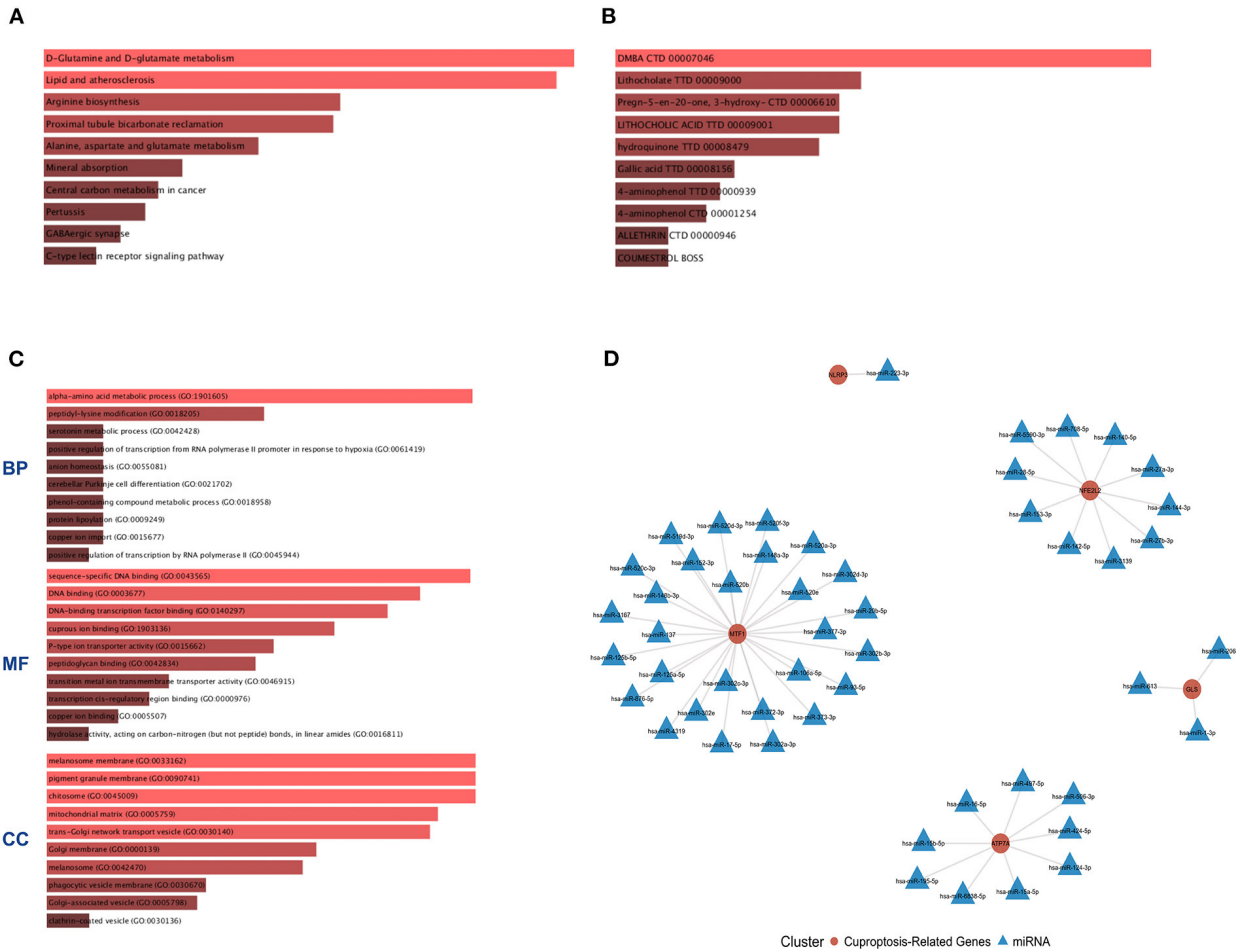
GO and KEGG pathway enrichment analysis of cuproptosis-related genes and drug target prediction. **(A)** KEGG analysis. **(B)** Drug target prediction. **(C)** GO analysis of the following components: BP, biological process; CC, cellular component; and MF, molecular function. **(D)** miRNA–mRNA network diagram of cuproptosis-related genes.

The six cuproptosis-related genes with a significant correlation with immune infiltration were included in the risk study. Based on binary logistic regression analysis, nomograms were constructed to calculate the total score to analyze the risk probability of the IS group (Figure 5B). The ROC curve (AUC = 0.9177) showed that nomograms had a good predictive ability for the assessment of risk factors in the IS group (Figure 5C). In addition, the calibration curve showed that the nomogram results were in good agreement with the actual results (Figure 5D). We also used GSE22255 as an external dataset to validate the gene signature, obtaining an AUC of 0.8142 (Supplementary Figure S2). Finally, the Enrichr website was used to enrich these six genes for GO analysis (Figure 6C) and KEGG pathway analysis (Figure 6A). In addition, drug targets were predicted (Figure 6B). The results of GO analysis indicated that genes were mainly enriched in biological processes (BPs), including alpha-amino acid metabolic process and so on. Molecular functions (MFs) analysis revealed that the genes were significantly enriched in equence-specific DNA binding, DNA binding, and DNA binding and so on. As for the cellular components (CCs), the genes were enriched in melanosome membrane and other processes and so on (Table 1). Regarding the results of KEGG pathway analysis as shown in Table 2, the genes in the up-regulated genome were primarily enriched in the pathways in D-Glutamine and D-glutamate metabolism and Lipid and atherosclerosis, etc. For drug target prediction, DMBA CTD 00007046, Lithocholate TTD 00009000 and other candidate molecules were predicted to have potential therapeutic effects because they had the highest odds ratio and the highest comprehensive score (Table 3). Finally, we constructed the IS cuproptosis-related DEG/miRNA–mRNA network and utilized FunRich software to predict the upstream miRNAs of cuproptosis-related DEGs. In total, 51 miRNAs, including hsa-miR-223-3p, hsa-miR-27a-3p and hsa-miR-28-5p, were obtained. Finally, the miRNA–mRNA regulatory network was visualized (Figure 6D).

**Table 1:** The GO function enrichment analysis of six cuproptosis related genes.

**Ontology**	**ID**	**Description**	***p* value**	**Odds ratio**	**Combined score**
BP	GO:1901605	Alpha-amino acid metabolic process	7.72E-05	226.7045	2,146.7751
BP	GO:0061419	Peptidyl-lysine modification	4.12E-04	95.6250	745.4340
MF	GO:0043565	Sequence-specific DNA binding	4.17E-04	41.1030	319.8792
MF	GO:0003677	DNA binding	0.0012	27.4006	194.9795
MF	GO:0140297	DNA-binding transcription factor binding	0.0016	23.7450	159.4630
CC	GO:0033162	Melanosome membrane	0.0036	363.3273	2,044.8828
CC	GO:0090741	Pigment granule membrane	0.0036	363.3273	2,044.8828
CC	GO:0045009	Chitosome	0.0036	363.3273	2,044.8828

**Table 2:** The KEGG function enrichment analysis of six cuproptosis related genes.

**Description**	***P*-value**	**Odds ratio**	**Combined score**
D-Glutamine and D-glutamate metabolism	0.0015	999.5000	6,499.5590
Lipid and atherosclerosis	0.0017	46.4343	296.7497

**Table 3:** The DSigDB analysis of six cuproptosis related genes.

**Description**	***P*-value**	**Odds ratio**	**Combined score**
DMBA CTD 00007046	1.00E-06	276.6944	3,821.3633
Lithocholate TTD 00009000	6.81E-06	832.5833	9,904.8578
Pregn-5-en-20-one, 3-hydroxy- CTD 00006610	7.86E-06	768.5000	9,032.6164
LITHOCHOLIC ACID TTD 00009001	7.86E-06	768.5000	9,032.6164
Hydroquinone TTD 00008479	8.98E-06	713.5714	8,291.8197

### IS component typing and GSVA

**Figure 7:**
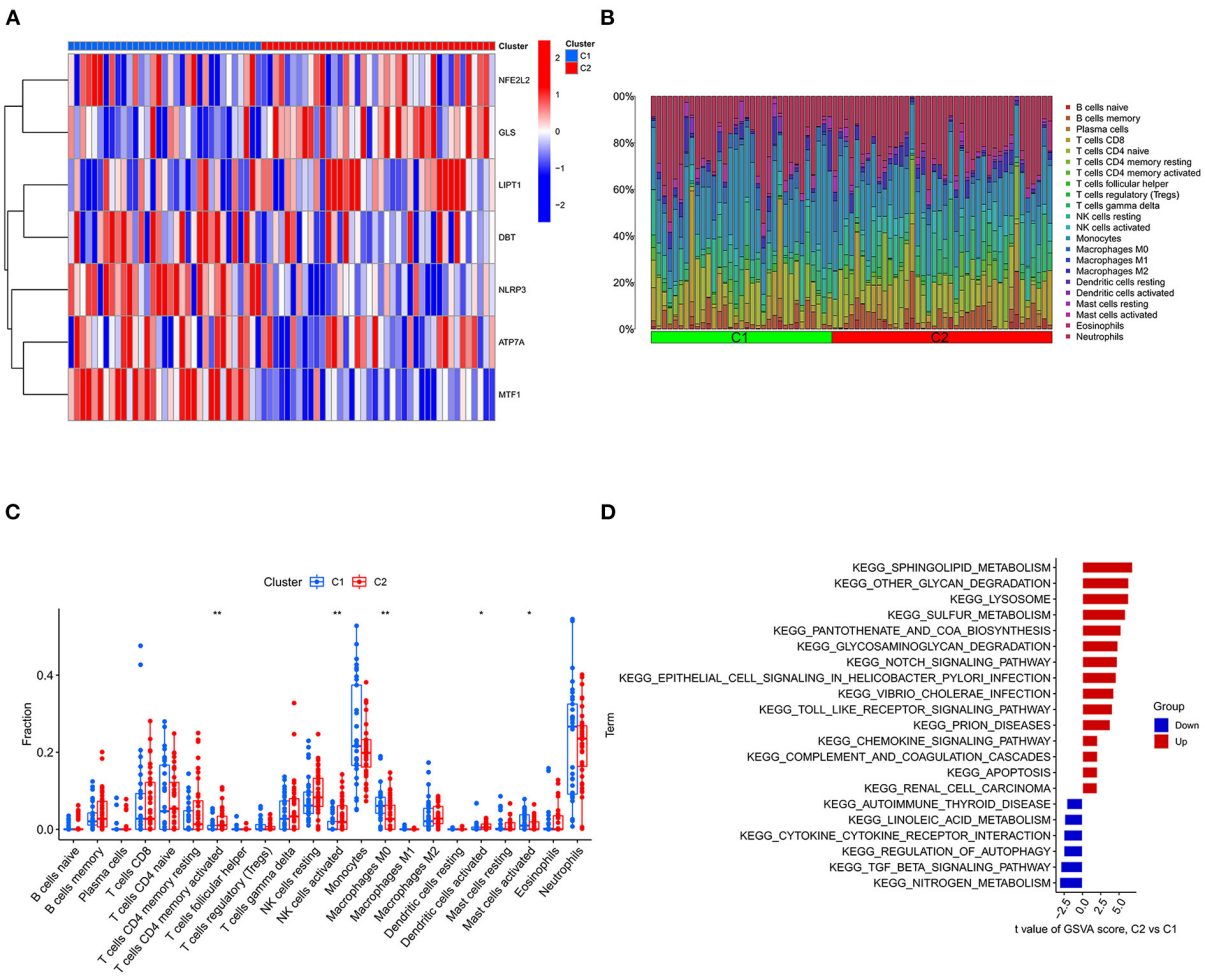
Typing and GSVA of the IS group samples. **(A)** Heatmap of the expression of cuproptosis-related DEGs in the two IS groups. The abscissa is C1 and C2, and the ordinate is cuproptosis-related DEGs. Blue represents low expression, and red represents high expression. **(B)** Relative percentages of 22 immune cells between the two subtypes. **(C)** Comparison of 22 immune cell subtypes between the two subtypes. **(D)** GSVA between the two subtypes. The vertical axis is the name of the function or pathway, and the horizontal axis is the test value. Red indicates that the biological function is upregulated in C2, and blue indicates that the biological function is upregulated in C1. *P < 0.05, **P < 0.01 and ***P < 0.001.

Consensus cluster analysis was performed using the ConsensusClusterPlus package in R software. According to the expression of cuproptosis-related DEGs, the optimal clustering stability was selected as k = 2 in consensus clustering (Supplementary Figure S1B). Therefore, 73 patients in the IS group were divided into two subgroups, namely, C1 and C2. PCA showed that the samples of C1 and C2 were well differentiated (Supplementary Figure S1D). Figure 7A shows a heatmap of the expression levels of cuproptosis-related DEGs in the two subtypes in the IS group. The expression of NLRP3 and MTF1 in C1 was higher than that in C2, and the expression of LIPT1 and GLS in C2 was higher than that in C1 (Supplementary Figure S1C). Therefore, the C1 group had high expression of cuproptosis-related genes. The relative percentages of 22 types of immune cells between the different types are shown in Figure 7B. Compared to C1, the number of activated memory CD4^+^ T cells, activated NK cells and activated dendritic cells was higher in C2. Compared to C2, the number of M0 macrophages and activated mast cells was higher in C1 (*p* < 0.05) (Figure 7C). Finally, GSVA was used to evaluate the difference in biological function between the different types. Figure 7D shows that the TGF-β signaling pathway and autophagy regulation pathways were upregulated in C1. Moreover, pathways, such as sphingolipid metabolism and other glycan degradation, were upregulated in C2.

## Discussion

Cuproptosis is a recently discovered form of cell death, which is characterized by intracellular free copper accumulation and protein lipidation, leading to cytotoxic stress and ultimately cell death (7). However, the mechanism of cuproptosis in IS has not been investigated. In the present study, microarray datasets were downloaded from the GEO database, and the correlation and difference in immune infiltration were analyzed. The results were then integrated with CC-related genes to construct a risk prediction model. Six cuproptosis-related genes were screened out, namely, NLRP3, NFE2L2, ATP7A, LIPT1, GLS and MTF1.

NFE2L2, also known as Nrf2, is a transcription factor involved in the regulation of various pathways, including heme/iron metabolism, metal ion regulation, antioxidant regeneration, glutathione regeneration, thioredoxin and intracellular protein recycling (16). Nrf2 plays a protective role in IS. Tanaka et al. (17) reported peak Nrf2 activity in the peri-infarct region 8 h after transient middle cerebral artery occlusion (tMCAO) in mice and decreased Nrf2 activity 24 h after tMCAO, and they also reported that the expression of antioxidant proteins downstream of Nrf2, such as heme oxygenase 1, glutathione and thioredoxin, is increased 24–72 h after tMCAO. Liu et al. (18) found that brain edema, neuronal death, inflammation and neurobehavioral defects are significantly reduced when NrF2-inducing drugs are administered 2–4 h after MCAO in mouse and rat models. Nrf2 participates in maintaining mitochondrial redox homeostasis by providing reduced glutathione and regulates Nrf2 activity through mitochondrial proteins such as DJ-1, PGAM5, and frataxin. A mutually regulatory loop forms between Nrf2 and mitochondria. Experimental observations on isolated mitochondria from Nrf2 knockout mice suggest that the role of Nrf2 in maintaining mitochondrial membrane integrity may involve a transition in mitochondrial membrane permeability. Nrf2 knockout cells exhibit increased spontaneous apoptosis and are highly sensitive to chemically induced mitochondrial damage, whereas activation of Nrf2 by chemoprotectants protects against mitochondrial damage (19).

NLRP3 has been extensively studied in IS. Previous studies demonstrated that the expression of NLRP3 protein significantly increased 24 and 72 h after tMCAO. At 72 h, the expression of NLRP3 protein reached its peak. From the 5th day after tMCAO, the expression of NLRP3 protein starts to decrease compared with before (20). Another study demonstrated that curcumin inhibits microglia/macrophage pyroptosis by inhibiting NF-κB and NLRP3 inflammasome, thereby improving white matter injury in ischemic stroke (21). ROS production, potassium efflux, mitochondrial disorder and lysosome rupture are currently recognized mechanisms of NLRP3 inflammasome activation. In a study of BV2 microglia, Xu et al. (22) reported that hypoxia and reoxygenation induce NLRP3 inflammasome activation through ROS, while activation of the Nrf2/ARE pathway activation inhibits NLRP3 inflammasome activation. Savage et al. found that after cerebral ischemia caused by middle artery occlusion in mice, IL-1 production by glial cells contributes to the increase in IL-6 and C-X-C motif chemokine 1 levels, the release of cathepsin B, and the induction of an inflammatory response. Therefore, the NLRP3 inflammasome is likely to induce the inflammatory response of cerebral ischemia–reperfusion injury through microglia (23). In the present study, NLRP3 was positively correlated with monocytes, while NLRP3 was negatively correlated with eosinophils. Previous studies have found that monocytes play a role in the process of atherosclerosis by activating the NLRP3 inflammasome (24). However, additional studies are required to fully understand how NLRP3 functions through monocytes in cerebral ischemia. Nrf2 and NLRP3 play a role in cerebral ischemia–reperfusion injury at the same time. Xu et al. (25) found that BAK ameliorates cerebral ischemic injury by inhibiting NLRP3-mediated inflammation through activating the Nrf2 signaling pathway. In the present study, the genetic correlation analysis found a strong correlation between Nrf2 and NLRP3. The present study showed that ROS/NF-κB was associated with the first step of NLRP3 inflammasome activation and that ROS/TXNIP and P2X7R were associated with the second step of NLRP3 inflammasome activation. Nrf2 is an upstream molecule of ROS and participates in both the first and second steps of NLRP3 inflammasome activation. Nrf2 alleviates cerebrovascular disease by inhibiting NLRP3 inflammasome activation (26). Mitochondrial dysfunction and the release of mtROS and mtDNA into the cytosol are other key upstream events related to NLRP3 activation. Mitophagy is an important regulator of NLRP3 activation, which can remove damaged and dysfunctional mitochondria and reduces mtROS. Furthermore, Nrf2-driven gene transcription attenuates NF-κB activation and downregulates the expression of inflammasome components NLRP3, CASP1, IL1B, and IL18, thereby limiting NLRP3 inflammasome activity (27).

NLRP3 is involved in various cell death pathways, including apoptosis, necroptosis, and ferroptosis. BAX and BAK are two essential intrinsic apoptotic effectors. Activation of the NLRP3 inflammasome is induced by promoting oxidized mitochondrial DNA (mtDNA) release and caspase-3/7-dependent K+ efflux in fever with thrombocytopenia syndrome (SFTS) virus (SFTSV) infection, DNA damage, chemotherapy, or cellular stress. DNA damage, chemotherapy, or cellular stress. Necrotic effectors contribute to the activation of the NLRP3 inflammasome. Mixed-lineage kinase domains such as pseudokinase (MLKL) are key executors of necrosis, promoting activation of the NLRP3 inflammasome by inducing K+ efflux (28). Ferroptosis is a new type of PCD. Recent studies demonstrated that NLRP3 inflammasome deficiency reduces cerebral ischemia-reperfusion injury by inhibiting ferroptosis, which may be achieved through the mechanism of the Keap1-Nrf2 pathway (29). Nrf2 regulation participates in genes that prevent ferroptosis, and under homeostatic conditions, NRF2 is ubiquitinated and targeted for proteasomal degradation by the KEAP1-CUL3-RBX1 E3 ubiquitin ligase complex. Under oxidative/electrophilic stress conditions, or due to mutations in KEAP1, CUL3, or Nrf2 itself, Nrf2 is no longer degraded, allowing nuclear translocation and activation of genes containing antioxidant response elements (ARE). Nrf2 transcriptional targets mediate iron/metal metabolism, catabolism/detoxification of reactive intermediates, and glutathione synthesis and metabolism, all of which play vital roles in preventing ferroptosis initiation (30). Drugs targeting both are also expected to be used to treat IS.

MTF1 is an important hypoxia-sensitive transcription factor. After tMCAO, remote limb ischemic postconditioning can trigger the translocation of MTF1 to the nucleus to bind to the metal response element, activate its transcription and induce the upregulation of NCX1 (31). Mtf1 has been characterized as a mitochondrial transcription factor and shown to regulate mitochondrial transcription. In fission yeast, MTF1 binds to Hsp60 *in vivo* and *in vitro*, and ETB inhibits the association of MTF1 and Hsp60, which represses mitochondrial transcription (32). The present study found that MTF1 had a significant positive correlation with mast cell activation. Therefore, we hypothesized that MTF1 may function through mast cells in IS.

Lipoic acid is a key co-factor of the 2-ketoacid dehydrogenase and glycine cleavage system in mitochondria, which is involved in the TCA cycle, mitochondrial energy metabolism, and amino acid catabolism. Lipoyltransferase 1, encoded by LIPT1, transfers lipoic acid to the E2 subunit of 2-ketoacid dehydrogenase. Thus, the absence of LIPT1 homologs may result in reduced E2 subunit lipidation. LIPT1 deficiency inhibits TCA cycle metabolism (33, 34). In recent years, LIPT1 has been reported to be a gene that may be associated with good prognosis in patients with urothelial carcinoma (35). LIPT1 overexpression in bladder cancer cell lines inhibits cell migration to some extent but has no effect on cell viability. We found that LIPT1 was downregulated in cerebral infarction and negatively correlated with NLRP3. The regulatory mechanism of LIPT1 on IS needs to be explored in the future.

Copper transport ATPase α (ATP7A) and β (ATP7B) are homologous P-type ATPases that use energy to pump copper across membranes, and they are ion-gated channels essential for cellular and systemic copper homeostasis. ATP7A controls translocation across the intestinal mucosa and BBB (36). ATP7A is a multilocus transmembrane protein encoded on the X chromosome. Menkes disease is a neurological disorder caused by mutations in ATP7A, an X-linked gene. Patients with this condition show marked neurological and developmental impairments due to disrupted copper delivery to the brain, resulting in copper deficiency in the brain, which leads to neuronal degeneration (37). Further experiments are needed to confirm whether ATP7A plays a role in the pathogenesis of IS.

In humans, glutaminase exists in two isoforms, namely, kidney-type (GLS) and liver-type (GLS2), and glutamine metabolism plays an important role in the normal metabolism of cells and in providing energy for rapidly proliferating cells and tissues (38). GLS-mediated breakdown of glutamine and its downstream production of α-ketoglutarate have been found to be critical for regulating the release of extracellular vesicles during HIV-1 infection and immune activation in neuroinflammation (39). Glutaminase catalyzes the breakdown of glutamine in mitochondria and regulates oxidative phosphorylation, redox state, and cellular metabolism to promote tumor growth. Furthermore, glutamine and glutamate, the substrate and product of the glutaminase reaction, can serve as signaling molecules to regulate redox and bioenergetic pathways in cancer (40). Although there are few studies on the mechanism of cuproptosis-related genes in the immune regulation of IS, based on previous studies and the results of this study, it can be speculated that cuproptosis-related genes may play an important role in the immune infiltration of IS. Further research is required to understand how to regulate the copper homeostasis of immune cells to prevent and treat IS.

Combining the immune infiltrating difference analysis results and the relevance of cuproptosis infiltration, we found that in ischemic stroke patients, the gamma delta T cells, monocytes, M0 macrophages and infiltrating neutrophils increased but that the infiltration of naive B cells, CD8^+^ T cells, regulatory T cells (Tregs) and NK cells decreased. Neutrophils are among the first cells to respond to ischemic brain injury. Neutrophils are involved in brain injury after cerebral ischemia by releasing proteases, including elastase, metalloproteinase (MMP9) and cathepsin G, as well as reactive oxygen species, nitrogen species and inflammatory IL-1β (41). After acute cerebral ischemia, monocytes migrate from *in situ* to the ischemic area, accumulate in the ischemic area and then differentiate into macrophages (42). Gamma delta T cells are a small fraction of T cells. After the onset of ischemic stroke, gamma delta T cells release IL-17a, IL-21, IL-22 and IFN-γ after activation and then participate in pathogenic processes, including the secretion of proinflammatory factors, BBB integrity and the recruitment of inflammatory cells into affected tissues, eventually leading to irreversible brain damage (43). CD8^+^ cytotoxic T cells are the first T cell subset to invade the ischemic brain and are detected within hours after stroke (44). In the present study, we found a lower proportion of CD8^+^ T cells in patients with ischemic stroke than in controls. However, whether the number of CD8^+^ T cells in peripheral blood samples reflects their pattern of infiltration in the vessel wall remains unclear. Most studies have demonstrated the protective effect of Tregs against ischemic stroke. Studies have found that Tregs regulate the poststroke immune response through neutrophils in the peripheral immune system, and Tregs inhibit the activation of T cell-mediated brain injury by effector T cells. Tregs also protect the BBB by interacting with C-C chemokine receptor type 5 (45). Most studies have shown that NK cell dynamics in IS are characterized by increased brain blood and decreased peripheral blood. Studies have found that NK cells are involved in immunosuppression and secondary infection after cerebral ischemia (46). Although there are few studies on the mechanism of cuproptosis-related genes in the immune regulation of IS, based on previous studies and the results of this study, it can be speculated that cuproptosis-related genes may play an important role in the immune infiltration of IS.

In addition, we also predicted pathways and drug targets that several cuproptosis-related genes may be involved with through the Enrichr database. We found that these six genes were mainly involved in two processes as follows: lipids and atherosclerosis; and D-glutamine and D-glutamate metabolism. Atherosclerosis is an important pathogenesis of acute ischemic stroke (47). Other studies have confirmed that abnormal neurological function during cerebral ischemia leads to increased release of the nerve excitatory amino acid, glutamate (Glu), resulting in significant increases in the concentration of Glu outside of the brain cells. With the surge in concentration, Glu excitotoxicity further promotes brain tissue damage, produces long-lasting excitatory postsynaptic potentials or causes calcium overload of nerve cells, leading to apoptosis and death of nerve cells (48). Through drug target prediction, DMBA CTD 00007046, Lithocholate TTD 00009000 and others were predicted to have potential therapeutic effects. By using IS-related microarray datasets and bioinformatics technology, we not only analyzed the correlation of immune infiltration and their differences between the IS group and the normal group but also analyzed the correlation between cuproptosis-related genes and immune cells and functions, providing information for the correlation between IS immunity and the cuproptosis mechanism. However, the gene-targeted drugs for IS treatment need to be confirmed by further research.

We predicted the upstream miRNAs of cuproptosis-related genes by FunRich software, and a total of 51 related miRNAs were obtained. The increase in biological sequencing data is beneficial for the further development of subsequent biological experiments to predict the association between noncoding RNAs and diseases through bioinformatics analysis (49). Prediction and quantification of the association between human noncoding RNAs and diseases through big data analysis effectively identifies the most relevant RNA diseases for experimental verification, thereby reducing the time and cost of biological experiments (50).

Further bioinformatics analysis of cuproptosis-related DEGs in IS samples revealed that the TGF-β signaling pathway and autophagy regulation pathways were upregulated in the subgroup with high expression of cuproptosis-related genes. TGF- β is an effective immunosuppressive agent. Disorders of TGF-β signaling are related to autoimmunity, inflammation and cancer, and TGF-β can mediate ERK, JNK and other classical pathways (51). The lack of oxygen and glucose supply caused by cerebral ischemia leads to an increased AMP/ATP ratio, which activates the AMPK pathway to initiate the autophagy process. Autophagy plays an important role in the pathogenesis of cerebral ischemia (52). Therefore, these highly expressed cuproptosis-related genes may contribute to the progression of acute ischemic stroke by mediating these pathways.

## Conclusions

In conclusion, we found that NLRP3, NFE2L2, ATP7A, LIPT1, GLS and MTF1 may serve as predictors of cuproptosis and play important roles in the pathogenesis of immune infiltration in IS. These results have a certain reference value for the subsequent basic research of cuproptosis in IS immunity. However, the present study l had some limitations. First, although the microarray data used met the sample size required for the study, there may be a certain bias in the results due to the small sample size. Second, although IS-associated cuproptosis-related genes were screened, their specific mechanism of action was not elucidated, indicating that subsequent studies are needed in the future.

## Data availability statement

The original contributions presented in the study are included in the article/Supplementary material, further inquiries can be directed to the corresponding authors.

## Author contributions

XF and HC performed experiments, wrote the manuscript, and conceived the idea of the study. YW, CX, and HW designed experiments. ML, WW, and JS analyzed the data. GL and DZ revised the final manuscript. All authors contributed to the article and approved the submitted version.

## Supplementary material

Supplementary Figure S1 **(A)** Distribution of cuproptosis-related genes on chromosomes. The outer circles represent chromosomes, and the names of the genes are annotated on the chromosomes. **(B)** The IS was divided into C1 and C2 according to the expression of cuproptosis-related DEGs. **(C)** Cuproptosis-related DEGs in different types (^**^*P* < 0.01 and ^***^*P* < 0.001). **(D)** PCA distinguished C1 and C2 samples based on the expression levels of cuproptosis-related DEGs.

Supplementary Figure S2 Receiver operating characteristic curves for the gene signature in the validation set of GSE22255.
