# Supplementary figures and images for: Comprehensive analysis of cuproptosis-related genes in immune infiltration in ischemic stroke
Source: Front Neurol. 2023 Feb 2;13:1077178. doi: 10.3389/fneur.2022.1077178 (PMC9933552; doi:10.3389/fneur.2022.1077178)

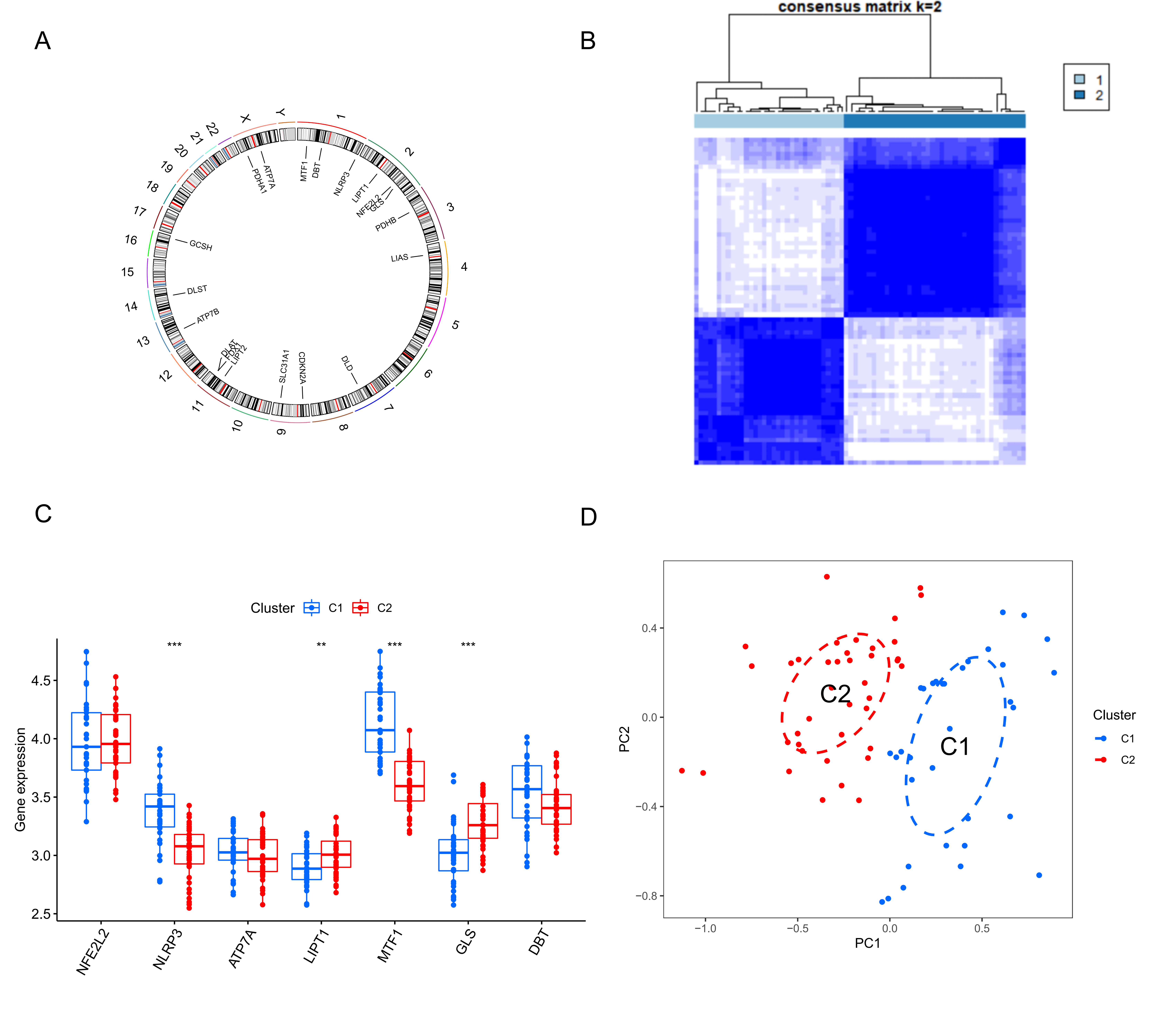

Supplement: Supplementary Figure S1 — (A) Distribution of cuproptosis-related genes on chromosomes. The outer circles represent chromosomes, and the names of the genes are annotated on the chromosomes. (B) The IS was divided into C1 and C2 according to the expression of cuproptosis-related DEGs. (C) Cuproptosis-related DEGs in different types (**P < 0.01 and ***P < 0.001). (D) PCA distinguished C1 and C2 samples based on the expression levels of cuproptosis-related DEGs. [file Image_1.TIF]

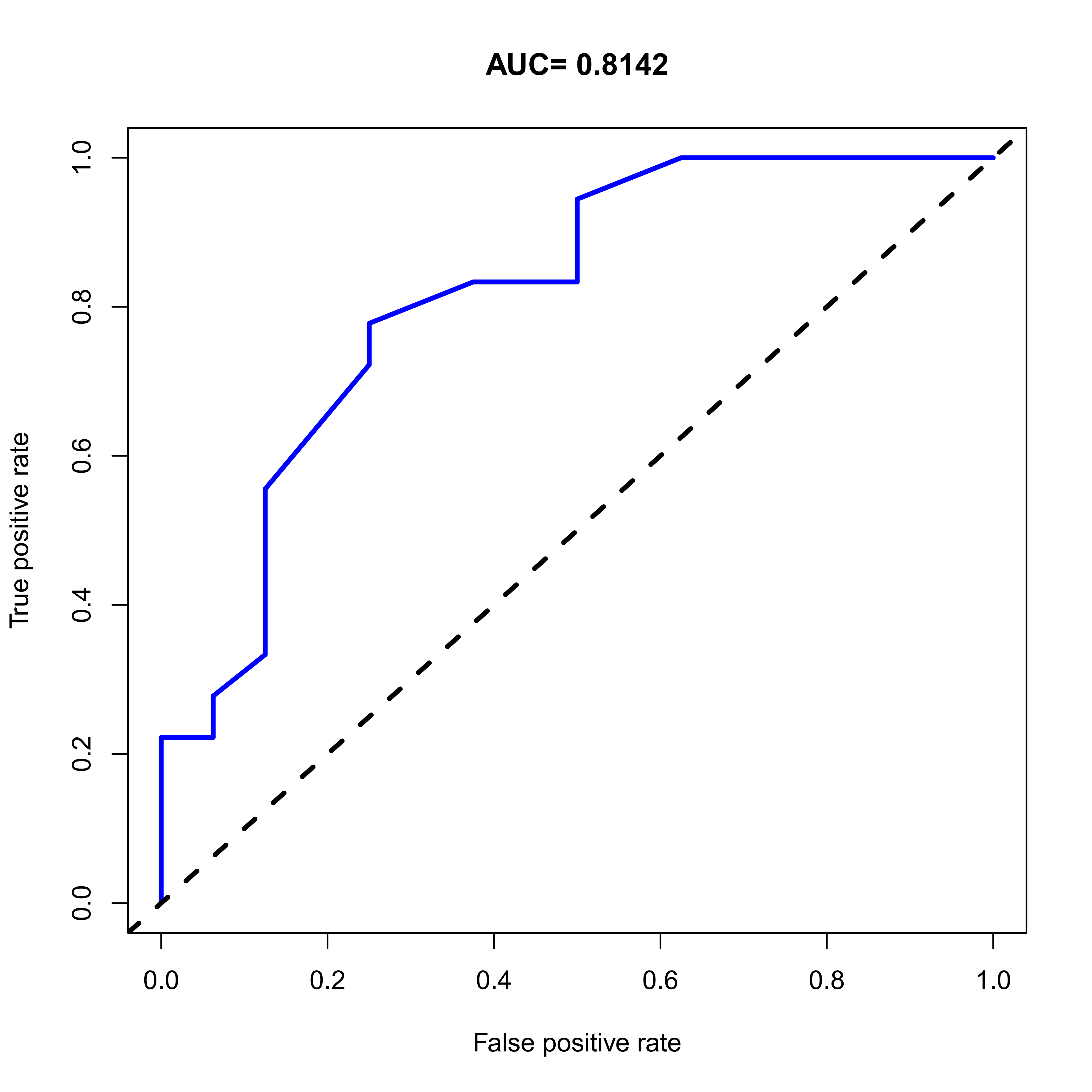

Supplement: Supplementary Figure S2 — Receiver operating characteristic curves for the gene signature in the validation set of GSE22255. [file Image_2.TIF]
