# Supplementary material for: Comprehensive analysis of cuproptosis-related genes in immune infiltration in ischemic stroke
Source: Front Neurol. 2023 Feb 2;13:1077178. doi: 10.3389/fneur.2022.1077178 (PMC9933552; doi:10.3389/fneur.2022.1077178)
Supplement: Supplementary file 3 [file Table_1.docx]

Supplementary Table. Cuproptosis-related Genes

| NFE2L2 |
| --- |
| NLRP3 |
| ATP7B |
| ATP7A |
| SLC31A1 |
| FDX1 |
| LIAS |
| LIPT1 |
| LIPT2 |
| DLD |
| DLAT |
| PDHA1 |
| PDHB |
| MTF1 |
| GLS |
| CDKN2A |
| DBT |
| GCSH |
| DLST |
